# *Rosellinia necatrix* infection induces differential gene expression between tolerant and susceptible avocado rootstocks

**DOI:** 10.1371/journal.pone.0212359

**Published:** 2019-02-14

**Authors:** Adela Zumaquero, Elsa Martínez-Ferri, Antonio J. Matas, Bianca Reeksting, Nicholas A. Olivier, Fernando Pliego-Alfaro, Araceli Barceló, Nöelani van den Berg, Clara Pliego

**Affiliations:** 1 Department of Genomics and Biotechnology, Fruticultura Subtropical y Mediterránea (IFAPA) Unidad Asociada de I+D+i al CSIC, Málaga, Spain; 2 Department of Crop Ecophysiology, Fruticultura Subtropical y Mediterránea (IFAPA) Unidad Asociada de I+D+i al CSIC, Málaga, Spain; 3 Department of Plant Physiology, Instituto de Hortofruticultura Subtropical y Mediterránea “La Mayora” (IHSM-UMA-CSIC), Unidad Asociada IHSM-IFAPA, University of Malaga, Málaga, Spain; 4 Department of Biochemistry, Genetics and Microbiology, University of Pretoria, Pretoria, South Africa; 5 Forestry and Agricultural Biotechnology Institute (FABI), University of Pretoria, Pretoria, South Africa; 6 Department of Plant and Soil Science, University of Pretoria, Pretoria, South Africa; ICAR-Indian Institute of Agricultural Biotechnology, INDIA

## Abstract

*Rosellinia necatrix* is the causal agent of avocado white root rot (WRR). Control of this soil-borne disease is difficult, and the use of tolerant rootstocks may present an effective method to lessen its impact. To date, no studies on the molecular mechanisms regulating the avocado plant response towards this pathogen have been undertaken. To shed light on the mechanisms underpinning disease susceptibility and tolerance, molecular analysis of the gene’s response in two avocado rootstocks with a contrasting disease reaction was assessed. Gene expression profiles against *R*. *necatrix* were carried out in the susceptible ‘Dusa’ and the tolerant selection BG83 avocado genotypes by micro-array analysis. In ‘Dusa’, the early response was mainly related to redox processes and cell-wall degradation activities, all becoming enhanced after disease progression affected photosynthetic capacity, whereas tolerance to *R*. *necatrix* in BG83 relied on the induction of protease inhibitors and their negative regulators, as well as genes related to tolerance to salt and osmotic stress such as aspartic peptidase domain-containing proteins and gdsl esterase lipase proteins. In addition, three protease inhibitors were identified, glu protease, trypsin and endopeptidase inhibitors, which were highly overexpressed in the tolerant genotype when compared to susceptible ‘Dusa’, after infection with *R*. *necatrix*, reaching fold change values of 52, 19 and 38, respectively. The contrasting results between ‘Dusa’ and BG83 provide new insights into the different mechanisms involved in avocado tolerance to *Phytophthora cinnamomi* and *R*. *necatrix*, which are consistent with their biotrophic and necrotrophic lifestyles, respectively. The differential induction of genes involved in salt and osmotic stress in BG83 could indicate that *R*. *necatrix* penetration into the roots is associated with osmotic effects, suggesting that BG83’s tolerance to *R*. *necatrix* is related to the ability to withstand osmotic imbalance. In addition, the high expression of protease inhibitors in tolerant BG83 compared to susceptible ‘Dusa’ after infection with the pathogen suggests the important role that these proteins may play in the defence of avocado rootstocks against *R*. *necatrix*.

## Introduction

Avocado (*Persea americana* Mill.), a member of the Lauraceae, is an important fruit crop in over 50 countries. The documented health benefits of avocado consumption are numerous, and avocado is considered one of the top 15 healthiest foods according to surveys across the United States and Western Europe (http://www.medicalnewstoday.com/articles/245259.php). There has been an increase in consumption worldwide during the last decade, bringing a certain concern about the difficulties of satisfying the demand in the near future. In this regard, efforts to increase avocado production by decreasing the incidence of avocado diseases are important. Significant losses in avocado production result from root rots caused by soilborne pathogens such as *Phytophthora cinnamomi* (*Phytophthora* root rot; PRR) and *Rosellinia necatrix* (white root rot; WRR). While *P*. *cinnamomi* affects avocado plantations worldwide, infections caused by *R*. *necatrix* have a considerable impact on avocado orchards in temperate regions, particularly Europe and Asia; however, recent out-breaks of this pathogen in other areas, such as California, México, Korea and South Africa, have increased the interest in gaining basic knowledge of its interaction with avocado.

To date, most investigations have focused on the control of PRR, and at present, an integrated approach involving the use of phosphate, tolerant rootstocks, and proper field management is giving positive results [1, 2]. Currently, there are commercial rootstocks with partial tolerance/resistance to *P*. *cinnamom*i (‘Thomas’, ‘Duke 7’ and ‘Dusa’), and significant efforts are in progress to select material with enhanced tolerance and other agronomic traits [3]. In addition, investigations are underway to gain a better knowledge of the avocado-*P*. *cinnamomi* interaction with the objective of developing tools that could be used to speed up avocado rootstock breeding and selection programmes. Along this line, Reeksting et al. [4] evaluated the transcriptomic responses through microarray analysis of the commercial rootstock ‘Dusa’ to *P*. *cinnamomi* and flooding, reporting the expression of many defence-related transcripts related to *P*. *cinnamomi* tolerance.

In contrast to PRR, the control of WRR disease remains a complex and difficult task due to the pathogen’s features, including resistance to drought, survival capacity in acidic soils, colonization of numerous hosts, deep penetration into the soil, and immunity to various common fungicides [5–7]. *R*. *necatrix* simultaneously invades avocado at several points within the roots, and invasion is followed by the proliferation of hyphal strands in one or several directions, colonizing both epidermal and cortical cells and finally, collapsing the vascular system of the plant [8].

Current control approaches involve the use of physical and chemical methods [9], although none have proven to be fully effective. In this sense, the utilization of tolerant/resistant rootstocks may represent an effective approach to lessen the impact of this soil-borne pathogen. To date, there are no commercial tolerant avocado rootstocks to *R*. *necatrix*, and tolerant selections to PRR, such as ‘Dusa’ or ‘Duke 7’, have been shown to be highly susceptible to this pathogen under artificial inoculation [5]. To overcome this problem, the biotechnology group at IFAPA-Málaga has been involved since 1995 in a breeding programme aimed at obtaining material with tolerance/resistance to WRR. In this programme, seeds collected from different provenances worldwide as well as asymptomatic trees located in areas affected by the pathogen have been evaluated [10]. To date, several selections showing tolerance to this fungus have been identified, and they are currently being tested under field conditions prior to commercialization. These selected genotypes provide a very useful tool for the study of the mechanisms involved in the avocado tolerant response to *R*. *necatrix*. In a previous study, Martinez-Ferri et al. [11] reported the physiological changes that occur during the initial stages of *R*. *necatrix* infection on avocado roots prior to the appearance of visible aboveground symptoms. While leaf photochemistry was affected at early stages of disease development in ‘Dusa’ (tolerant to PRR, but susceptible to WRR), even when no aerial symptoms were visible, no changes could be detected in the tolerant BG83 genotype (one of the selections from IFAPA-Málaga breeding programme tolerant to WRR), despite the presence of the fungus in the root system. The absence of changes at the photosynthetic level in the BG83 genotype suggested that the tolerance to *R*. *necatrix* could be associated with mechanisms conferring a higher robustness to the photosynthetic machinery of the plant.

Examining avocado gene expression profiles in contrasting disease reactions will contribute to a better understanding of the molecular interactions underlying tolerance and susceptibility to *R*. *necatrix* and would allow the identification of new genes that could be used as markers in avocado rootstock breeding programmes. Therefore, the aim of this work was to analyse the transcriptome responses of tolerant (BG83) and susceptible (‘Dusa’) avocado rootstocks to *R*. *necatrix* by using an Agilent array in which genes involved in the incompatible response to *P*. *cinnamomi* and flooding are represented. Since some of the upregulated genes in the avocado-*P*. *cinnamomi* interaction have already been identified [12, 13], this study will also shed light on the differences at the transcriptome expression level between the pathogens *P*. *cinnamomi* and *R*. *necatrix* when infecting avocado.

## Results and discussion

### Post-infestation development of physiological and aerial symptoms

**Fig 1:**
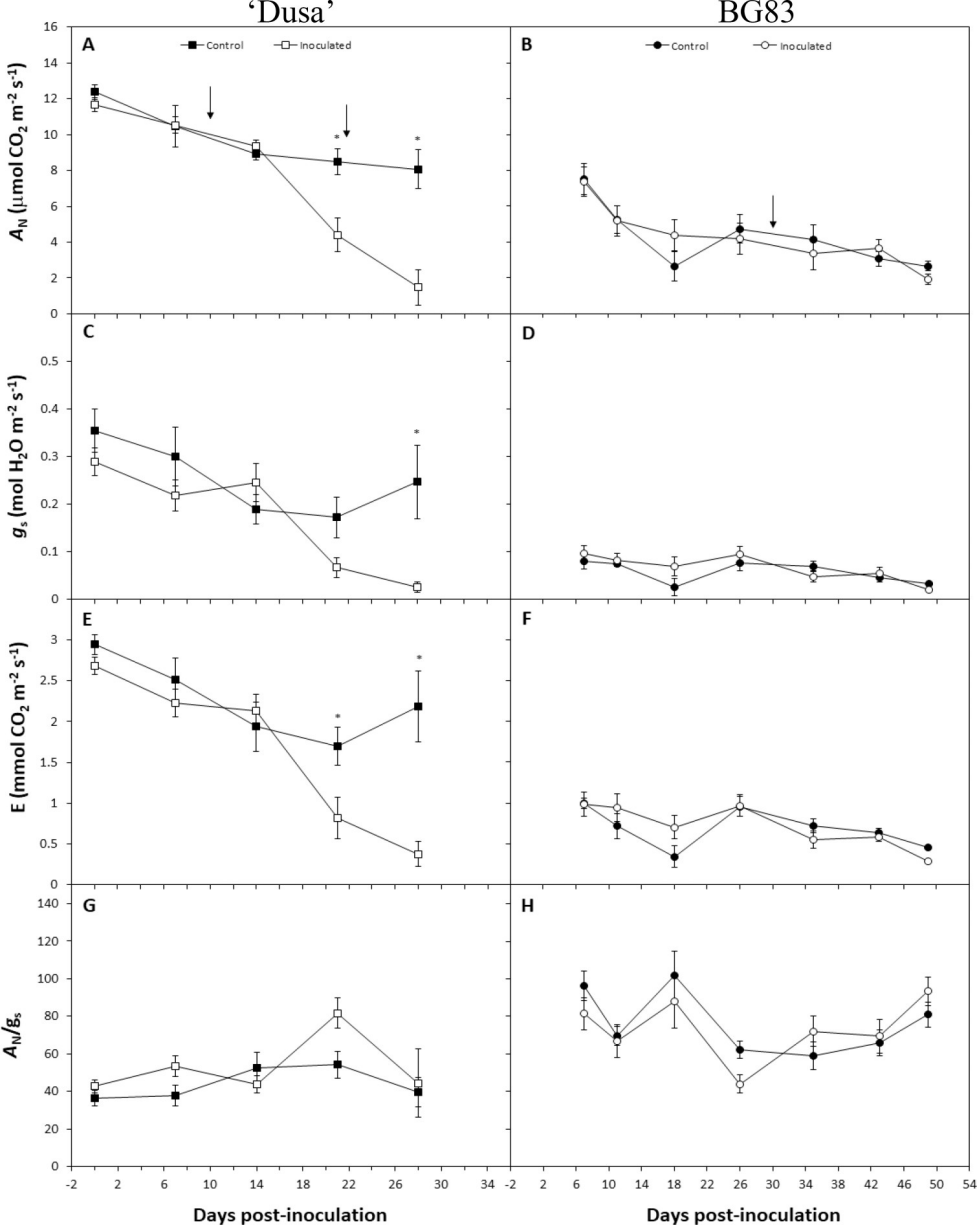
Post-infestation development of physiological and aerial symptoms. Variation in net assimilation rates (*A*_N_; A and B), stomatal conductance (*g*_s_; C and D), transpiration rate (*E*; E and F) and intrinsic water use efficiency (*A*_N_/*g*_s_; G and H) in ‘Dusa’ and BG83 avocado plants at stage 1 (no aerial symptoms) after inoculation with *R*. *necatrix*. The asterisks indicate significant differences between inoculated and control treatments (*P*<0.05). Each data point represents the mean (±SE; n = 8 to 18). The arrows indicate root sampling for RNA extractions.

The primary mechanisms of woody plants in response to root pathogen infections are associated with the impairment of water relations and the limitation of photosynthesis [14, 15]. In a previous study, Martinez-Ferri et al. [11] showed that *R*. *necatrix* infection in susceptible avocado rootstocks caused a decrease in photosynthesis at the photochemical level, suggesting that pathogen infection could be associated with stomatal limitations of photosynthesis. In this regard, the effects of *R*. *necatrix* on photosynthetic performance during the first stages of disease progression by gas exchange measurements were analysed. The results showed an early and significant decrease in CO_2_ assimilation and a decline in stomatal conductance in the susceptible genotype ‘Dusa’ after 22 days post-inoculation prior to the appearance of any aerial symptoms. In this genotype, net assimilation rates (*A*_N_) decreased concomitantly with stomatal closure (*g*_s_) and transpiration rates (*E*), resulting in no variation in the instantaneous water use efficiency (Fig 1A, 1C, 1E and 1G). In contrast, no significant variation in any of the gas exchange parameters was observed during disease progression in the tolerant BG83 genotype (Fig 1B, 1D, 1F and 1H). This result indicates an effective ability of this genotype, either at the root or at the leaf levels, to counteract the pathogen’s effect on water relations, avoiding photosynthetic decline. This BG83 tolerant response to *R*. *necatrix* is different from the ‘Dusa’ tolerant response to *P*. *cinnamomi*, in which tolerance was associated with a greater ability to restore both *A*_N_ and *gs* at similar levels as those seen in the non-inoculated control [16]. In this sense, it is remarkable that BG83 displayed lower values of *A*_N_, *g*_s_, and *E* than ‘Dusa’ but higher *A*_N_/g_s_, suggesting a behaviour of avoidance of water losses [17], which is consistent with the higher robustness of the photosynthetic machinery previously reported by Martinez-Ferri et al. [11].

### Transcriptome responses of avocado

To analyse the avocado response to *R*. *necatrix* infection in tolerant and susceptible avocado, we used a targeted cDNA microarray containing transcripts from *de novo* sequencing of a *Phytophthora* tolerant ‘Dusa rootstock in response to flooding and *P*. *cinnamomi* infection [12]. Root samples of the susceptible ‘Dusa’ genotype infected with *R*. *necatrix* were collected before (‘Dusa’-b) and after (‘Dusa’-a) CO_2_ assimilation rates in the leaves significantly decreased (8 and 22 days post-inoculation, respectively). In contrast, root samples from the BG83 genotype were collected in asymptomatic infected plants eight days after the observation of a decrease in the net assimilation rate in the susceptible ‘Dusa’ genotype (30 days post-inoculation). The hybridization percentages of the microarrays were similar for the three samples at 55.6% for ‘Dusa’-b, 70.8% for ‘Dusa’-a and 61.9% for BG83.

**Fig 2:**
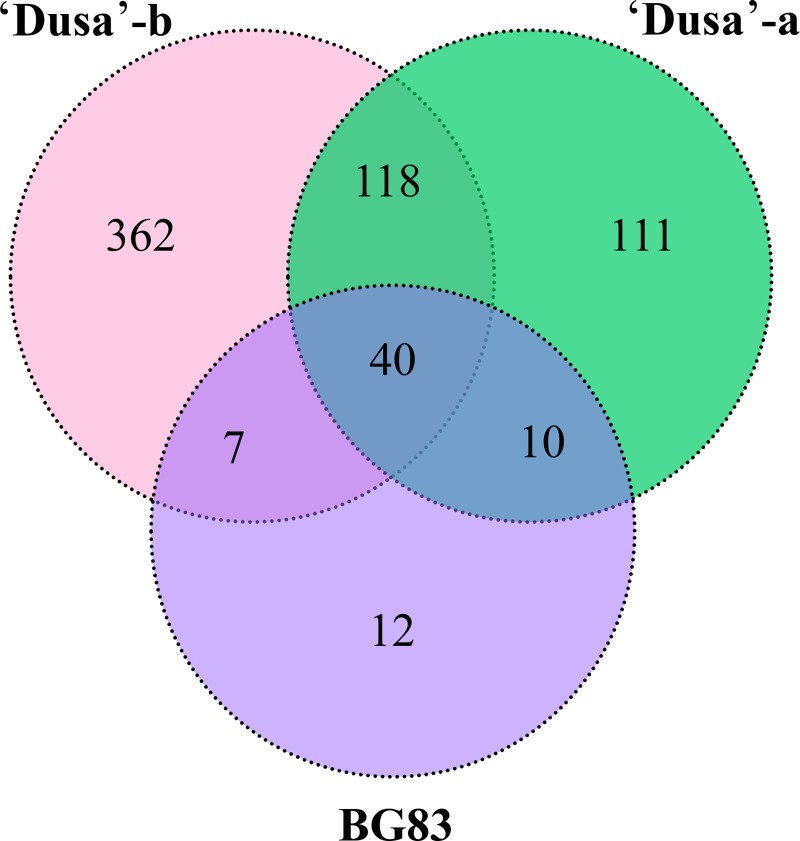
Venn diagram of differentially expressed genes. Numbers of common and specific differentially expressed genes (DEGs) obtained in the microarray analysis of susceptible ‘Dusa’-b, ‘Dusa’-a and tolerant BG83 avocado rootstocks after infection with *R*. *necatrix*. Shared transcripts are illustrated where the circles meet, while unique DEGs are shown in only one of the three circles. A higher number of both specific and shared DEGs, were observed in the susceptible ‘Dusa^’^ than in the tolerant BG83 rootstock.

The total number of genes on the array with significantly altered expression elicited by *R*. *necatrix* infection was 527 (80.4% induced and 19.6% repressed), 279 (76.6% induced and 23.4% repressed) and 69 (85.5% induced and 14.5% repressed) transcripts in ‘Dusa’-b, ‘Dusa’-a and BG83, respectively (Adj. P-value < 0.05). As shown in Fig 2, 362 genes were specific to ‘Dusa’-b and 111 to ‘Dusa’-a, while 12 genes were exclusively deregulated in BG83 (Fig 2). The lower number of genes with significantly altered expression observed in tolerant plants seems to be a consequence of a better performance during *R*. *necatrix* infection in comparison to sensitive plants and could be linked to a higher number of constitutively expressed genes in the tolerant genotype when compared to the susceptible one, in accordance with results obtained in other plant-pathogen interactions; e.g., olive genotypes susceptible to *Verticillium dahliae* showed significantly more upregulated differentially expressed genes (DEGs) than tolerant plants upon infection [18]. Similar results have also been observed in tolerant and susceptible sugarcane and rice genotypes used in other stress studies, e.g., water stress-sensitive plants activated their response mechanisms to water deficit earlier than the tolerant ones, showing a larger number of stress-responsive transcripts [19, 20].

### Validation of the microarray

Differences found in gene expression profiles between susceptible and tolerant rootstocks to *R*. *necatrix* were further verified by performing a real-time quantitative qPCR (qRT-PCR) assay on total cDNA samples from roots of three biological replicates. Fifteen unigenes showing contrasting expression patterns among Dusa-b, Dusa-a and BG-83 were analysed. This selection included three genes potentially involved in the tolerance of BG to *R*. *necatrix* (Pa_contig02540; Pa_contig05213; Pa_contig04097). Negative controls were used to confirm the absence of contamination. *Actin* was used as a reference gene for data normalization. The expression levels of these genes amplified by qRT-PCR are shown in Table 1. Although higher expression values were obtained by qRT-PCR than those observed on the microarray, the results corroborated the overall differences found among the three samples (‘Dusa’-b, ‘Dusa’-a, BG83) in the microarray analysis.

**Table 1:** qRT-PCR and microarray expression data of selected contigs from susceptible ‘Dusa’-b, ‘Dusa’-a and tolerant BG83 avocado rootstocks infected with *R*. *necatrix*.

**Name**	Annotation	qRT-PCR FC ‘Dusa’-b	Microarray FC ‘Dusa’-b	qRT-PCR FC ‘Dusa’-a	Microarray FC ‘Dusa’-a	qRT-PCR FC BG83	Microarray FC BG83
Pa_contig 00651	12-oxophytodienoate reductase 11	**387.99**	**14.49**	**403.39**	**10.57**	**26.85**	2.83
Pa_contig 03380	Conserved hypothetical protein	**379.27**	**6.11**	**137.06**	**3.76**	7.06	1.23
Pa_contig 00931	Receptor protein kinase ZmPK1	**137.29**	**7.94**	**97.86**	**6.71**	**5.75**	1.84
Pa_contig 02033	Glutamine amidotransferase	122.54	5.34	**50.73**	4.55	21.11	**4.96**
Pa_contig 03267	Peroxidase 5-like	117.16	**20.81**	18.15	**4.75**	5.35	*ND*
Pa_contig 05854	Protein hothead-like	20.12	**7.86**	**340.76**	**23.88**	367.94	**6.03**
Pa_contig 01404	Putative plant syntaxin	25.55	**5.20**	**57.32**	**12.26**	**4.08**	*ND*
Pa_contig 05917	Germin-like protein	**14.42**	**10.35**	**54.46**	**25.17**	0.50	*ND*
Pa_contig 02874	Germin-like protein subfamily 1 member 20-like	8.61	**17.28**	**22.88**	**30.04**	-0.49	*ND*
Pa_contig 00951	Kynurenine formamidase-like	**10.24**	*ND*	**12.85**	**12.98**	12.27	**6.13**
Pa_contig 05213	Glu proteasa inhibitor	13.94	**16.19**	**32.85**	*ND*	**52.07**	**43.66**
Pa_contig 02540	Tumor related protein	**28.67**	**8.18**	**31.86**	**22.06**	**38.31**	**16.01**
Pa_contig 04097	Trypsin inhibitor	6.18	**5.19**	-4.17	**2.75**	**18.80**	**33.80**
Pa_contig 00456	Methionine gamma-lyase-like	3.30	*ND*	**3.37**	*ND*	**4.44**	**6.43**
Pa_contig 04185	Defensin j1-2-like	-11.04	**-6.40**	-73.48	**-6.35**	-431.88	**-33.12**

The data are displayed as fold changes (FC), calculated by comparing plants inoculated with *R*. *necatrix* with control plants (non-inoculated). The expression data are the mean of three biological replicates. The numbers in bold indicate statistically significant results (*t*-test, P<0.05). *ND*: not detected.

### Tolerant and susceptible avocado rootstocks show divergent differentially expressed gene (DEG) patterns during root infection with *R*. *necatrix*

**Fig 3:**
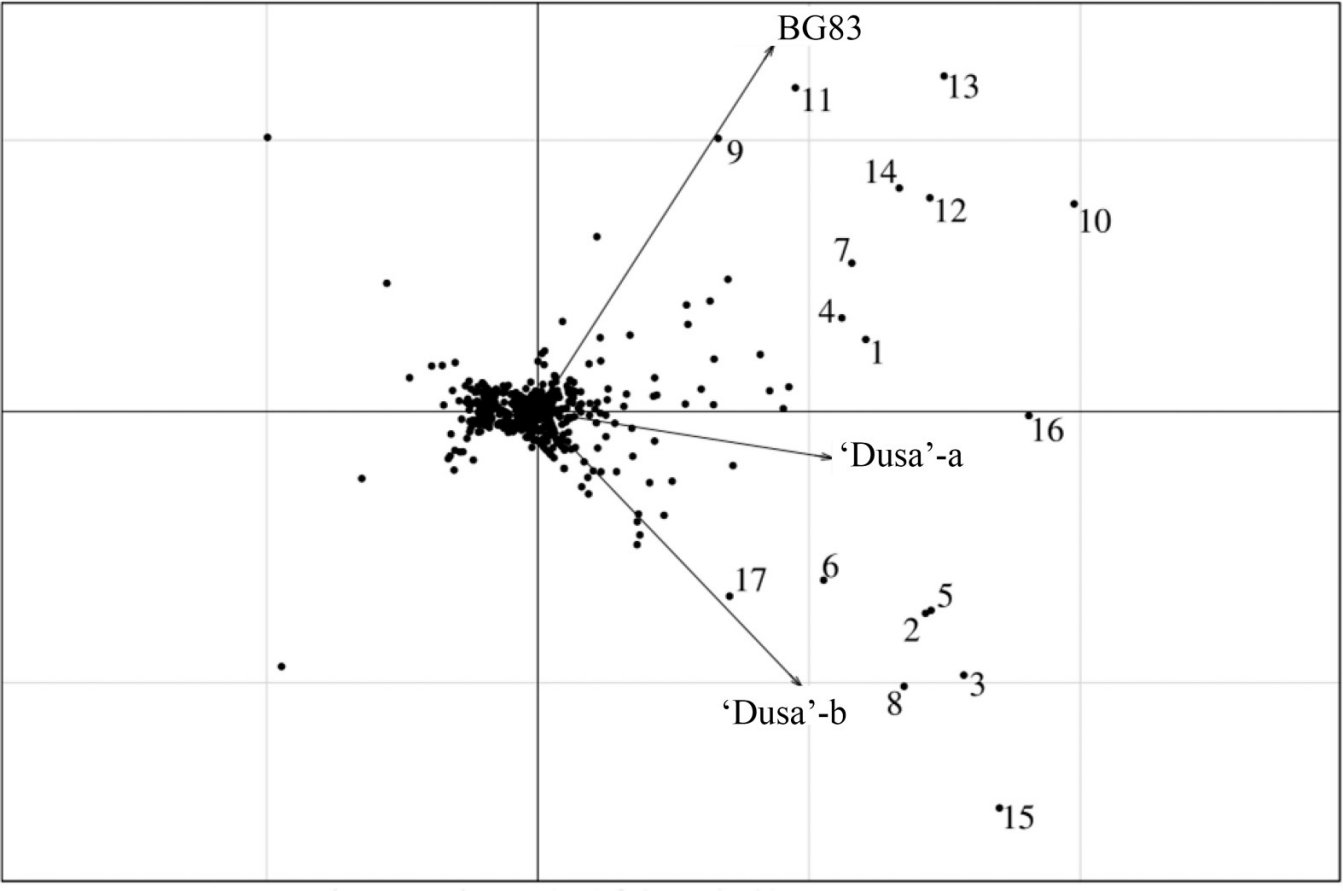
Principal component analysis (PCA) of differentially expressed transcripts (DEGs). PCA of DEGs obtained in the microarray analysis of susceptible ‘Dusa’-b, ‘Dusa’-a and tolerant BG83 avocado rootstocks after infection with *R*. *necatrix*. PCA of DEGs present in at least one of the samples, in which the first two dimensions explain up to 88.74% of the variance. Samples from the tolerant BG83 avocado rootstock showed the largest differences with those from the susceptible ‘Dusa’(‘Dusa’-b, ‘Dusa’-a), which were grouped together in the biplot. Numbers are as follows: **1**: protein hothead-like (Pa_Contig00205); **2**: chitinase 1-like (Pa_Contig00535); **3**: beta-glucanase (Pa_Contig00542); **4**: non-annotated (NA) (Pa_Contig00559); **5**: basic endochitinase-like partial (Pa_Contig01014); **6**: thaumatin-like protein (Pa_Contig01450); **7**: basic 7s globulin-like (Pa_Contig02817); **8**: glucan endo-beta-glucosidase-like (Pa_Contig03461); **9**: trypsin inhibitor (Pa_Contig04097); **10**: protein hothead-like (Pa_Contig04808); **11**: glu protease inhibitor-like (Pa_Contig05213); **12**: protein hothead-like (Pa_Contig06176); **13**: NA (Pa_Contig06358); **14**: basic 7s globulin-like (Pa_Contig06808); **15**: pathogenesis-related protein pr-4-like (Pa_Contig07140); **16**: protein hothead-like (Pa_Contig07184); **17**: NA (Pa_Contig07403).

Studies of different gene expression profiles occurring in roots of tolerant and susceptible avocado genotypes are crucial to explain the tolerance or susceptibility to *R*. *necatrix* in avocado rootstocks. In this study, gene expression profiles taking place in roots of the susceptible *versus* tolerant genotype were analysed and compared. Principal component analysis (PCA) was performed on all transcripts significantly modified by infection. The scores plot of the PCA showed a stronger relationship between ‘Dusa’-b and ‘Dusa’-a with BG83 being the most dissimilar sample (Fig 3).

**Fig 4:**
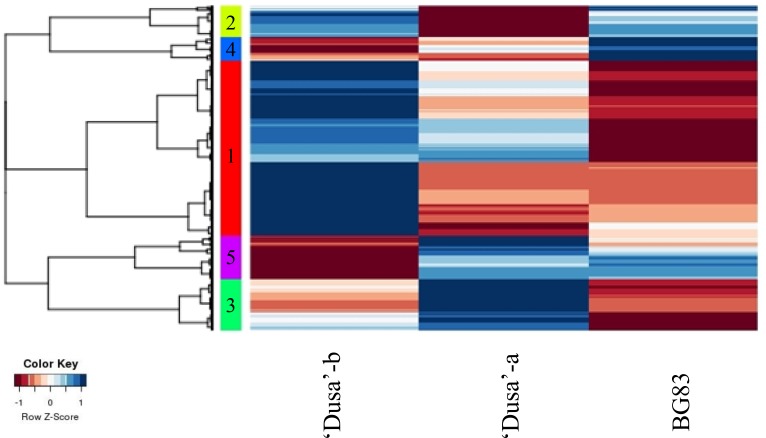
Hierarchical clustering (HCL) of differentially expressed genes (DEGs). HCL of DEGs in at least one of the samples of ‘Dusa’-b, ‘Dusa’-a and tolerant BG83 avocado rootstocks after infection with *R*. *necatrix*, clustered by hclust according to their expression profiles (Pearson’s values correlation analysis). The five colours on the left correspond to the five groups with different expression profiles.

Hierarchical clustering (HCL) was performed on DEGs according to the expression profiles obtained from the microarray (Fig 4); ‘Dusa’-b and ‘Dusa’-a showed closer expression patterns than those observed for the tolerant BG83 genotype in which a high percentage of the genes were repressed (red colour). Genes were clustered into five groups according to the expression pattern observed after *R*. *necatrix* infection (S1 Table). Group 1 (red in Fig 4) included DEGs that were predominantly induced in ‘Dusa’-b but not in BG83. This is the largest group, including 361 DEGs. The biological processes most commonly identified with GO-term-enriched analysis (P<0.025) comprised, among others, oxido-reduction processes, heme binding, recognition of pollen, hydrolase activities and iron ion binding (S1 Fig). Genes included in this group showing higher induction values were pathogenesis-related protein PR-4-like (Pa_contig07140), thaumatin-like protein 1 (Pa_contig01450), glucan endo-beta-glucosidase-like (Pa_contig03461) and beta-glucanase (Pa_contig00542). These genes, except for the one encoding the PR-4-like protein, are included in the top10 genes showing higher induction in ‘Dusa’-b and ‘Dusa’-a (Table 2), decreasing their expression once alterations of net assimilation rates at the leaf level were observed (‘Dusa’-a) and not detectable in BG83. These genes have been described as putative defence-related genes in ‘Dusa’/*P*. *cinnamomi* interactions, when ‘Dusa’ behaves as a tolerant rootstock [4, 12, 13], suggesting that they play an important role in the tolerance against *P*. *cinnamomi* but not against *R*. *necatrix* infection. This group also includes the top10 repressed genes in BG83 (Table 2), with a gene encoding a defensin-like protein (Pa_contig04185) showing the highest repression. This gene is also repressed in the susceptible cultivar but at lower levels. Although plant defensins are a group of pathogenesis-related defence mechanism proteins [21], their high repression in the tolerant cultivar indicates that they do not play a key role in tolerance to *R*. *necatrix*.

**Table 2:** Top 10 avocado transcripts differentially regulated by *R*. *necatrix* in ‘Dusa’-b, ‘Dusa’-a and BG83.

‘Dusa’-b			‘Dusa’-a			BG83		
Name	Description	FC	Name	Description	FC	Name	Description	FC
Pa_Contig07140	Pathogenesis-related protein pr-4-like.	119.12	Pa_Contig04808	Protein hothead-like.	78.74	Pa_Contig06358	—NA—	45.98
Pa_Contig03461	Glucanendo- -beta-glucosidase-like.	79.64	Pa_Contig07184	Protein hothead-like.	74.12	Pa_Contig05213	Glu protease inhibitor-like	43.66
Pa_Contig00542	Beta- -glucanase.	72.50	Pa_Contig01014	Basic endochitinase-like partial.	72.48	Pa_Contig04808	Protein hothead-like	37.54
Pa_Contig07403	Pathogenesis-related protein	56.50	Pa_Contig00535	Chitinase 1-like.	69.84	Pa_Contig06808	Basic 7s globulin-like (aspartic peptidase containing protein)	37.51
Pa_Contig01014	Basic endochitinase-like partial.	49.89	Pa_Contig00542	Beta-glucanase.	63.30	Pa_Contig04097	Trypsin inhibitor	33.80
Pa_Contig00535	Chitinase 1-like.	46.60	Pa_Contig00205	Protein hothead-like.	58.11	Pa_Contig06176	Protein hothead-like	29.39
Pa_Contig01450	Thaumatin-like protein.	45.00	Pa_Contig01450	Thaumatin-like protein.	47.88	Pa_Contig02817	Basic 7s globulin-like (aspartic peptidase containing protein)	21.66
Pa_Contig07184	Protein hothead-like.	43.16	Pa_Contig03461	Glucanendo- -beta-glucosidase-like.	41.21	Pa_Contig00559	—NA—	21.52
Pa_Contig01608	Cytochrome p450 89a2-like.	42.84	Pa_Contig06015	Beta-D-galactosidase.	41.00	Pa_Contig07184	Protein hothead-like	20.44
Pa_Contig01569	Peroxidase n1-like.	38.37	Pa_Contig06358	—NA—	40.20	Pa_Sin_HA66E9C01BVIYG	—NA—	19.41
Pa_Sin_GI32N0T02IWL2D	Hypothetical protein LEMA_P014390.1	-82.05	Pa_Contig02823	Uncharacterized protein LOC104613500	-12.62	Pa_Contig04185	Defensin j1-2-like	-33.12
Pa_Contig02835	Probable nucleoredoxin 2	-39.33	Pa_Contig04303	Metallothionein-like protein type 2	-10.73	Pa_Contig06047	—NA—	-12.59
Pa_Contig00540	Protein exordium-like 2	-19.30	Pa_Contig07016	Chalcone synthase	-10.25	Pa_Contig00062	Polyphenol chloroplastic-like	-7.46
Pa_Contig01573	Cytochrome p450 714c2-like	-18.58	Pa_Contig06047	—NA—	-9.53	Pa_Contig04951	Gibberellin-regulated protein 3	-6.95
Pa_Contig02105	Protein walls are thin 1-like	-13.96	Pa_NA_RC_Contig06966	—NA—	-9.12	Pa_Sin_HA66E9C01AIWJ3	Heat shock protein 21	-6.95
Pa_Contig03065	Protein gast1-like	-12.11	Pa_Contig01429	Dihydroflavonol 4-reductase	-7.87	Pa_Contig01098	21 kda protein	-6.06
Pa_Contig06047	—NA—	-9.32	Pa_NA_F_contig06354	—NA—	-7.79	Pa_NA_F_contig07407	—NA—	-5.90
Pa_Contig05365	Starch branching enzyme isoform 3	-8.19	Pa_Contig04354	Anthocyanidin reductase	-7.67	Pa_Contig00751	Xyloglucanendotransglucosylasehydrolasefamilyprotein	-5.74
Pa_Contig02780	Uncharacterized loc101208739 isoform 1	-7.99	Pa_Contig03042	Gibberellin-regulated protein 6	-7.64	Pa_Sin_GI32N0T02GTAO6	Glucose-1-phosphate adenylyltransferase large subunit 1-like	-5.43
Pa_Contig01427	Tubulin alpha chain	-7.90	Pa_Contig03065	Protein gast1-like	-7.31	Pa_Contig01026	Polyphenol chloroplastic-like	-3.28

NA: non annotated. FC: Fold Change.

Group 2 (yellow in Fig 4) consisted of 60 DEGs predominantly upregulated in ‘Dusa’-b and BG83 but not in ‘Dusa’-a. Among the genes included in this cluster, the most noticeable behaviour was for a glu-protease inhibitor like (Pa_contig 05213), a gene with much higher expression in BG83 when compared to ‘Dusa’, (FC BG83 = 43.66 and FC ‘Dusa’-b = 16.19 in the microarray analysis and FC BG83 = 52.07 and FC ‘Dusa’-b = 13.94 in qRT-PCR experiments). The role of protease inhibitors in plant defence against phytophagous insects and microorganisms has been extensively reported [22, 23]. Interestingly, eight out of the top10 repressed transcripts selected for ‘Dusa’-a are included in this group (Table 2); the metallothionein-like protein (Pa_contig04303) repressed in ‘Dusa’ after *R*. *necatrix* infection (Pa_04303) has been recently included within the top 25 avocado transcripts induced in an incompatible avocado/ *P*. *cinnamomi* interaction, highlighting the important role of this gene in tolerance to this oomycete [24], while its expression is not detected in the BG83 genotype, tolerant to *R*. *necatrix*. Genes whose expression was increased as the infection progressed in the susceptible genotype (repressed or slightly induced in ‘Dusa’-b and highly induced in ‘Dusa’-a) comprised groups 3 and 5 (green and purple, respectively, in Fig 4). These groups include 102 and 88 DEGs, respectively. The biological processes most commonly identified with GO-term-enriched analysis (P<0.025) in group 3 comprised mainly cell wall macromolecule catabolic processes, chitinase activity, chitin binding and chitin catabolic processes (S2 Fig). This is in accordance with the top10 differentially expressed transcripts selected for ‘Dusa’-b (Table 2), in which five of the genes are involved in cell wall catabolic processes (basic endochitinase Pa_contig01014, chitinase1 Pa_contig00535, beta-glucanase Pa_contig00542, glucan endo-beta-glucosidase Pa_contig03461 and beta-d-galactosidase Pa_contig06015). In contrast to glucanases, chitinases increase their expression as the disease progresses, and their role as defence-related proteins from the attack of pathogens has been widely described [25]. Expression of chitinase genes was also observed in tolerant rootstocks but at lower levels, suggesting constitutive rather than induced resistance mechanisms, as previously described in the *Humulus lupulus-Verticillium albo*-*atrum* interaction [26].

The smaller group comprising 50 genes, Group 4 (blue in Fig 4), includes those mainly induced in BG83. Many of these genes are related to an oxidation-reduction response and include defence-related genes such as cytochrome *p450*, protease inhibitors, and btb poz and taz domain-containing protein 1-like genes, which are known to play a role in plant defence [27–29].

**Fig 5:**
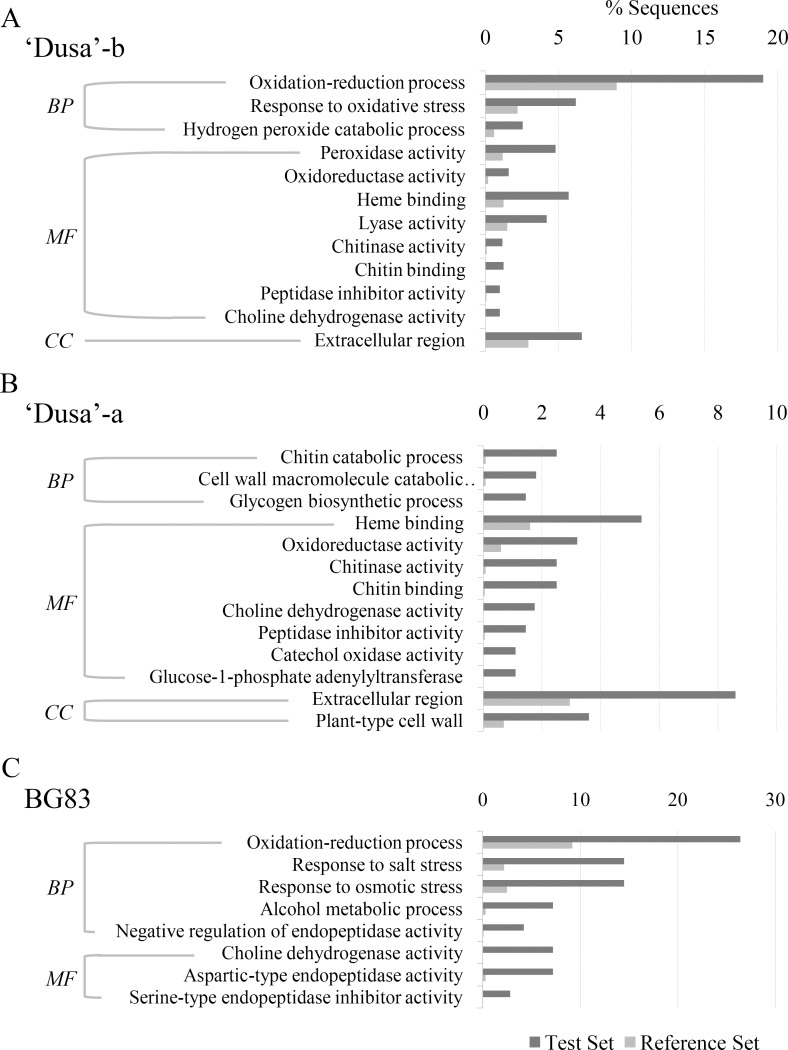
Gene Ontology (GO) enrichment analysis of differentially expressed genes (DEGs). GO enrichment analysis of DEGs in susceptible ‘Dusa’-b, ‘Dusa’-a and tolerant BG83 avocado rootstocks after infection with *R*. *necatrix*. Enrichment GO terms were obtained by Blast2GO using a cut-off of 0.025. (BP) biological process; (MF) molecular function; (CC) cellular component.

Subsequently, to identify processes and functions over-represented in the different genotypes, all differentially expressed genes were functionally enriched and categorized based on blast sequence homologies and GO annotations using Blast2GO software (P<0.025) (Fig 5). In relation to the susceptible genotype, we observed commonly responsive genes before and after a significant decrease in the CO_2_ assimilation rate in the leaves was detected; these genes were related to oxidoreductase activity, chitinase activity, chitin and heme binding, choline dehydrogenase activity and peptidase inhibitor regarding molecular function activity and associated with the extracellular region regarding cellular components. Some GO terms were specific to ‘Dusa’-b, such as oxidation-reduction processes, response to oxidative stress, hydrogen peroxide catabolic processes within biological process and peroxidase and lyase activities regarding molecular functions (Fig 5A). All of them had been induced in response to the reduction-oxidation status of the host cells as a result of pathogen invasion. In contrast, the most significant GO terms found solely in ‘Dusa’-a were related to cell wall macromolecule catabolic processes, chitin catabolic processes and glycogen biosynthetic processes included in biological processes; glucose-1-phosphate, adenylyl transferase and catechol oxidase activities included in molecular functions, and plant cell wall-type activities included in cellular components (Fig 5B), most of them probably related to cell wall degradation as a consequence of disease progression [26].

Seven GO terms were enriched in DEGs from the BG83 genotype, and two were also found in the susceptible genotype (oxidation-reduction process and choline dehydrogenase). Among the GO terms found solely in the tolerant genotype were the following: response to salt and osmotic stresses, alcohol metabolic process and negative regulation of endopeptidase activity, in biological processes; and aspartic-type endopeptidase activity and serine-type endopeptidase inhibitor activity, in molecular function.

The differential induction of genes involved in salt and osmotic stress in BG83, such as aspartic peptidase domain-containing proteins (Pa_contig06808, Pa_contig04419, Pa_contig02817, Pa_contig05393) [30], gdsl esterase lipase protein (Pa_contig00520) [31] and btb poz and taz domain-containing protein (Pa_contig00582) [32], could indicate that *R*. *necatrix* penetration into the roots is associated with osmotic effects, suggesting that BG83’s tolerance to *R*. *necatrix* is related to the ability to withstand osmotic imbalance. This is in agreement with the collapse of the vascular system and the diminished water flow towards the aerial parts of the plant reported after *R*. *necatrix* invasion of root epidermal and cortical cells [8, 11].

Other GO terms expressed solely in BG83 seem to be associated with controlled protein degradation, which has been reported to be essential for the growth and development of plants and for survival under abiotic and biotic stresses [33]. To date, several protease and protease inhibitors have been confirmed to have functions in plant defence against pathogens [34, 35]. In addition, other studies reported that protease inhibitors also play a role in tolerance to abiotic stress, such as salt stress. Li et al. [36] showed that transgenic plants expressing a cysteine protease inhibitor exhibited increased salinity resistance, better growth performance, lower malondialdehyde contents, higher anti-oxidase activity, and higher cell viability under salinity stress. Hence, it appears that tolerance to salt and osmotic stress, control of protein degradation and tolerance to *R*. *necatrix* share common pathways which merit further investigations.

### Differential expression of defence-related genes in ‘Dusa’ and BG83 avocado rootstocks after infection with *R*. *necatrix*

Genes previously reported to be important for conferring resistance in the ‘Dusa’-defence response against *P*. *cinnamomi* were shown to be upregulated during ‘Dusa’-*R*. *necatrix* interaction (Table 3). Some of them, such as the protein PR-4-like, cytochrome p450, thaumatin-like protein, glutathione S-transferase and chitinases, were even included in the list of the 20 genes showing the highest expression in ‘Dusa’ after infection with *R*. *necatrix* (S2 Table).

**Table 3:** Defence-related genes overexpressed in ‘Dusa’-b, ‘Dusa’-a and BG83 avocado rootstock following *R*. *necatrix* infection.

Contig ID ‘Dusa’-b	Contig ID ‘Dusa’-a	Contig ID BG83	Annotation
Pa_contig00582	Pa_contig00582	Pa_contig00582	Btb poz and taz domain-containing protein 1-like
Pa_contig02574			Universal stress protein a-like protein
Pa_contig00410			Phenylalanine ammonia-lyase (PAL)
Pa_Sin_GI32N0T02IMW			Lipoxygenase (LOX)
Pa_contig01014, Pa_contig06246	Pa_contig00472, Pa_contig01014, Pa_contig01395, Pa_contig06246, Pa_contig00472	Pa_contig01395	Endochitinase
Pa_contig01422, Pa_contig01568		Pa_contig01568	Glutathione S-transferase
Pa_contig01288, Pa_contig01450, Pa_contig01462	Pa_contig01288, Pa_contig01450, Pa_contig01462	Pa_contig01462	Thaumatin-like protein 1
Pa_contig01608, Pa_contig02847, Pa_contig03623, Pa_contig05603, Pa_contig06747, Pa_contig07325, Pa_contig07667	Pa_contig02847	Pa_contig01652, Pa_contig07667	Cytochrome p450
Pa_contig01236, Pa_contig05917	Pa_contig01236, Pa_contig02874, Pa_contig05917		Germin-like protein
Pa_contig00371, Pa_contig00528, Pa_contig01007, Pa_contig01569, Pa_contig03234, Pa_contig04589, Pa_contig05459, Pa_contig06331, Pa_Sin_FZ03KKT01A7ZOH, Pa_Sin_GI32N0T02F7BG6, Pa_Sin_GI32N0T02GDB8Q, Pa_Sin_GI32N0T02GS0JQ, Pa_Sin_GI32N0T02H6JSO, Pa_Sin_GI32N0T02HAYGM, Pa_Sin_GI32N0T02HMZMX, Pa_Sin_GI32N0T02HQGBT, Pa_Sin_GI32N0T02I1757	Pa_contig00371, Pa_contig03890, Pa_contig05459, Pa_Sin_GI32N0T02HAYGM, Pa_Sin_GI32N0T02HMZMX, Pa_Sin_GI32N0T02HQGBT, Pa_Sin_GI32N0T02I1757, Pa_Sin_GI32N0T02JURYY, Pa_Sin_HA66E9C01BY5PD	Pa_contig01569, Pa_contig05459	Peroxidase
Pa_contig02540, Pa_contig04097, Pa_contig05213, Pa_Sin_HA66E9C01AKZ67	Pa_contig02540, Pa_contig04097, Pa_Sin_HA66E9C01AKZ67	Pa_contig02540, Pa_contig04097, Pa_contig05213	Protease inhibitor
Pa_contig6278			Pathogenesis-related protein 4 (PR-4)
Pa_contig05982, Pa_contig07140, Pa_Sin_HA66E9C01BLTDJ	Pa_contig01063, Pa_contig05982, Pa_contig07140		Pathogenesis-related protein (PR)

Five endochitinases were induced in ‘Dusa’-b, two of them (basic endochitinase-like partial and acidic endochitinase-like) showing FC values of 49.89 and 11.34, respectively. Only one endochitinase (endochitinase pr4-like) was expressed in the tolerant rootstock BG83 (Table 3). An increase in the transcript levels of endochitinases was reported in tolerant avocado rootstocks, including ‘Dusa’, during the first 24h of infection with *P*. *cinnamomi* [13]. Chitinases belong to the PR protein families PR3, 4, 8 and 11 and are involved in the plant defence response against pathogens. The higher upregulation of chitinase genes in ‘Dusa’ compared to BG83 could be a consequence of a higher fungal colonization of susceptible plant cells and subsequent activation of fungal cell-wall degradation.

Inoculation of ‘Dusa’ and BG83 avocado plants with *R*. *necatrix* resulted in an accumulation of P450-like transcripts. Cytochrome P450 monooxygenases (P450s) are ubiquitous enzymes that catalyse the oxidation of many substrates by the activation of molecular oxygen and are involved in biotic and abiotic environmental responses as well as in the HR response to infection [37, 38, 39]. Different P450-like proteins were induced in susceptible and tolerant genotypes, e.g., while seven P450-like proteins were highly induced in the ‘Dusa’ genotype, only two were upregulated in BG83 (Table 2).

Expression of thaumatin-like protein (PR5) was only observed in ‘Dusa’ after infection with *R*. *necatrix*. PR5 has been reported to be associated with the salicylic acid (SA) pathway, and it has also been described to be significantly upregulated in ‘Dusa’ 48h after *P*. *cinnamomi* infection [13]. The fact that PR5 is upregulated in both ‘Dusa’-*R*. *necatrix* and ‘Dusa’-*P*. *cinnamomi* interactions, suggests that its induction must be important for tolerance to *P*. *cinnamomic* but not to *R*. *necatrix*. These results agree with previous studies reporting that SA signalling is mostly associated with defence against biotrophs, while regulation by ethylene and jasmonic pathways is a major defence response against necrotrophic pathogens [37, 40]. This is consistent with the significant overexpression of genes regulated by jasmonate, such as btb poz and taz domain-containing protein 1-like, observed in the tolerant rootstock BG83 (Table 2). Future studies addressing the transcriptome analysis of *R*. *necatrix*-tolerant rootstock interaction will be needed to confirm the expression of genes associated with the jasmonate or ethylene pathways.

Peroxidases are PR9 proteins induced in plant tissues upon pathogen infection and are known as defence-related proteins. In avocado roots, they seem to play a role in counteracting massive production of reactive oxygen species, which are induced when *P*. *cinnamomi* invades root cells [41]. In this study, *R*. *necatrix* infection induced 21 peroxidase genes in susceptible ‘Dusa’ and only two in the tolerant BG83 genotype. Low peroxidase expression in tolerant genotypes has also been described in other studies, i.e., rice genotypes in response to *Fusarium fujikuroi* [37, 42], which has been attributed to a reduced spread of the pathogen.

Plant protease inhibitors are extensively studied for their role in defence against pests and pathogens due to their ability to inhibit specific proteases of the intruder [43] and are used to develop transgenic plants resistant to pathogens [44]. In this study, we identified three protease inhibitors (Pa_contig05213; Pa_contig02540; Pa_contig04097) (Table 1) that were highly overexpressed in the tolerant BG83 genotype when compared to susceptible ‘Dusa’, after infection with *R*. *necatrix*, reaching FC values of 44, 34 and 16, respectively, in microarray analysis and FC values of 52.7, 18.8 and 38.31, respectively, in qRT-PCR experiments. Two of these protease inhibitors (Pa_contig05213 and Pa_contig02540) have been shown to be upregulated in tolerant ‘Dusa’ after 18 h post-inoculation with *P*. *cinnamomi*, suggesting an important role of these proteins in the tolerance of avocado to the oomycete [24].

Interestingly, trypsin inhibitor overexpressed in buckwheat seeds suppressed spore germination and mycelial growth in the phytopathogenic fungi *Alternaria alternata* and *Fusarium oxysporum* [45]. The expression of these protease inhibitors is more than 20 times higher in tolerant BG83 compared to susceptible ‘Dusa’ after infection with the pathogen, suggesting the important role that these proteins could play in the defence of avocado rootstocks against *R*. *necatrix*. Since protease inhibitors are widespread in plant tissues and highly active with respect to proteases of pathogenic insects, bacteria and fungi [35, 44], they should be taken into consideration in plant breeding programmes aiming to improve plant resistance.

### Comparative model of *Rosellinia necatrix* infection on susceptible ‘Dusa’ and tolerant BG83 genotypes

**Fig 6:**
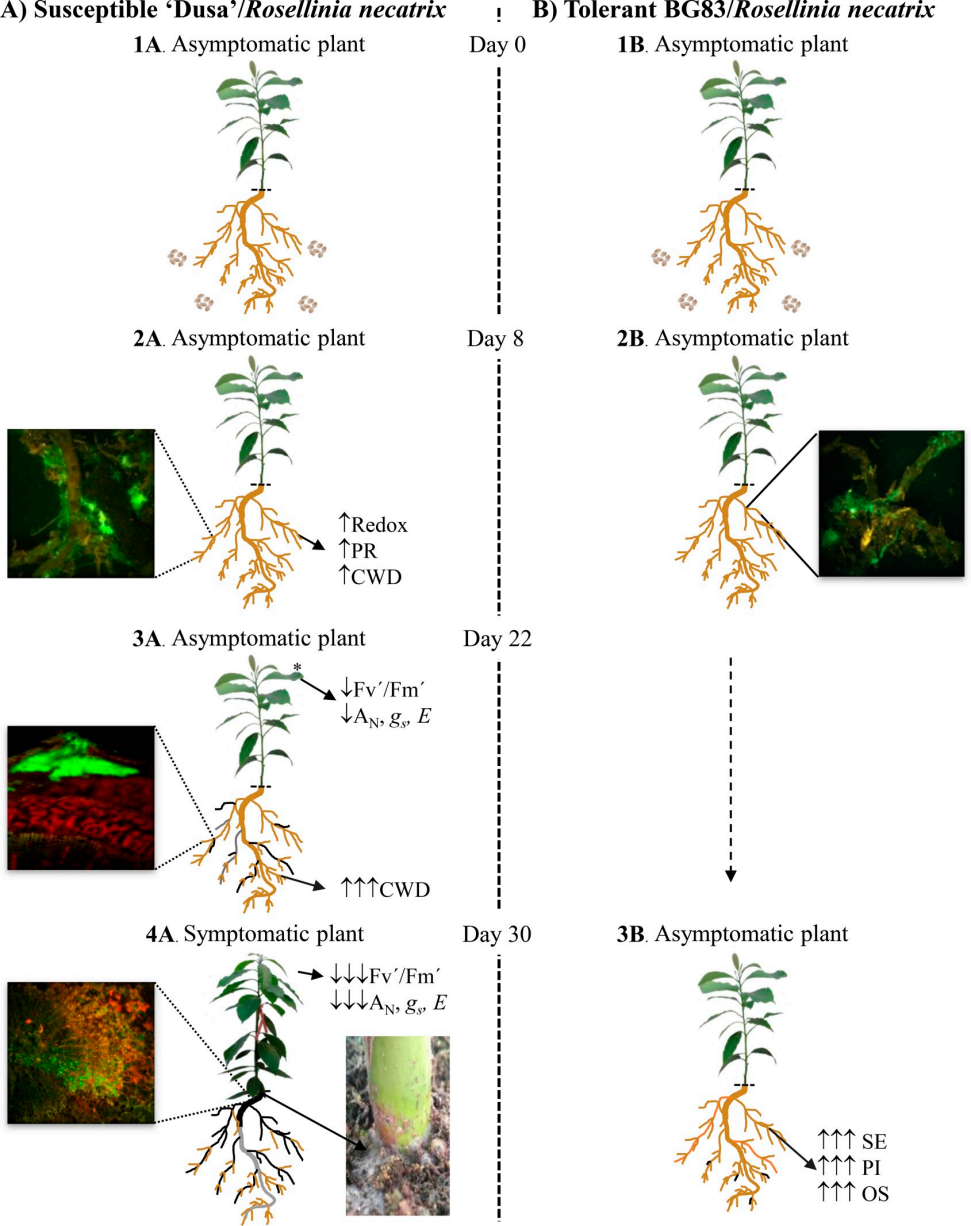
**Comparative model of *Rosellinia necatrix* infection on susceptible** ‘**Dusa**’ **(A) and Tolerant BG83 genotypes (B)**. **1A**. Inoculation of asymptomatic ‘Dusa´plant with wheat grains infected with *R*. *necatrix*. **2A**. 8 days post inoculation. Visualization of *R*. *necatrix* derivative strain CH53-*gfp* mycelia colonizing the root surface of avocado plantlets without aerial symptoms. The green fluorescent emitted from *R*. *necatrix*-*gfp* was visualized using a fluorescence microscope. Upregulation of genes mainly related to oxidoreduction activity (Redox), cell wall degradation (CWD) as well as potential genes involved in defence to *P*. *cinnamomi* (PR) was observed. **3A**. 22 days post inoculation. The image shows a radial section of susceptible roots infected with *R*. *necatrix*-*gfp*; penetration and proliferation of *R*. *necatrix* mycelia through the root cortex was observed by Confocal Laser Scanning Microscopy (CLSM) concomitant with a decrease in leaf photochemistry (Fv´/Fm´) and gas exchange parameters (*A*_N_, *gs*, *E*). Expression of genes related with cell wall degradation enzymes (CWD) mainly, chitin related enzymes, become more relevant. **4A**. 30 days post inoculation, mild-wilting symptoms of the leaves start to appear in susceptible genotype. CLSM images of radial sections of the roots revealed a profuse invasion of *R*. *necatrix*, collapsing vascular vessels. **1B**. Inoculation of asymptomatic BG83 plant with wheat grains infected with *R*. *necatrix*. **2B**. 8 days post inoculation. Visualization of *R*. *necatrix* derivative strain CH53-*gfp* mycelia colonizing the root surface of avocado plantlets without aerial symptoms. **3B**. 30 days post inoculation. Asymptomatic tolerant BG83 genotype showing upregulation of genes related to osmotic (OE) and salt stress (SE) as well as genes encoding protease inhibitors (PI).

Taking into consideration previous studies [7, 8, 11] together with results presented in this investigation, a scheme of early disease progression in susceptible and tolerant avocado/*R*. *necatrix* interactions is proposed integrating molecular and physiological data as well as Confocal Laser Scanning Microscopy (CLMS) observations (Fig 6). Plants were inoculated with wheat grain infected with *R*. *necatrix* (Day 0). Eight days after inoculation, *Rosellinia necatrix* spread through the soil, reaching avocado roots (visualization under a fluorescence microscope Leica Microsystems using a derivative strain of *R*. *necatrix* expressing the green fluorescent protein, *R*. *necatrix*-*gfp*) [8, 11]. Leaf photochemistry was not affected, and no aerial symptoms were observed in any of the genotypes. Molecular data revealed that infected susceptible ‘Dusa’ showed an upregulation of genes mainly related to oxidoreduction activity, cell wall degradation (chitinases and glucanases) as well as potential genes involved in defence to *P*. *cinnamomi* (pathogenesis-related protein PR-4-like (Pa_contig07140), thaumatin-like protein 1 (Pa_contig01450), glucan endo-beta-glucosidase-like (Pa_contig03461) and beta-glucanase (Pa_contig00542)). After 22 days of inoculation, although no visible symptoms were observed in any of the genotypes, variation in trapping efficiency of photosystem-II (Fv´/Fm´) [11], net assimilation rates (*A*_N_), stomatal conductance (*g*_s_) and transpiration rate (*E*) were observed in susceptible ‘Dusa´. At the root level, genes related to cell wall macromolecule catabolic processes, chitinase activity, chitin binding and chitin catabolic processes became more relevant. CLSM observations using *R*. *necatrix*-*gfp* derivative strain, revealed that once leaf assimilation rates were affected in susceptible cultivars, external colonization of *R*. *necatrix* mycelia and penetration of mycelial aggregates into the root system followed by hyphal proliferation can be observed, although the root collar of the plant was not yet affected [11]. At thirty days post-inoculation, changes in net assimilation rates and leaf photochemistry became more pronounced, and visible symptoms, such as mild wilting of the leaves, started to appear in ‘Dusa’. A profuse invasion of the vascular system and fungal colonization of the root collar was observed under CLSM studies, severely obstructing water flow to aerial parts of the plant [8].

At this stage, the tolerant genotype BG83 did not show either physiological changes or visible symptoms, although the fungus was present in the roots and penetration had occurred (visible and fluorescence microscopy observations). Molecular studies revealed an induction of protease inhibitors and their negative regulators, as well as salt- and osmotic stress-related genes.

## Conclusions

This study represents the first report on gene expression analysis for assessing the response of tolerant and susceptible avocado rootstocks to *R*. *necatrix*. The microarray analysis showed differential gene expression against this pathogen in tolerant and susceptible avocado rootstocks. In the susceptible genotype, the early response was mainly related to oxido-reduction processes and cell-wall degradation activities, which became more noticeable after the photosynthetic capacity was affected by disease progression. In contrast, in addition to oxido-reduction processes, tolerance to *R*. *necatrix* in BG83 relies on the induction of protease inhibitors and their negative regulators, as well as salt and osmotic stress-related genes.

Furthermore, this study provides new insights into the different mechanisms involved in avocado tolerance responses to *P*. *cinnamomi* and *R*. *necatrix*. Interestingly, *R*. *necatrix* induced in susceptible ‘Dusa’ a set of general defence-related transcripts (i.e., universal stress protein, thaumatin-like protein and cytochrome p450) that are also induced in the tolerant response of ‘Dusa’ to *P*. *cinnamomi*. Some of these genes with enhanced expression are related to salicylic acid-dependent pathways involved in the defence mechanism against hemi-biotrophic pathogens such as *P*. *cinnamomi*. These genes were not noticeable in BG83. However, in BG83, a high induction of protease inhibitors, which have been extensively associated with plant defence against pathogens and abiotic stresses such as water stress [43], was observed. These results could indicate that these protease inhibitors play an important role in avocado tolerance against *R*. *necatrix*. Since protease inhibitors have been reported to be induced by water stress, in future studies, we will address the possibility of enhancing avocado tolerance to *R*. *necatrix* by priming with water stress. In addition, further transcriptomic analysis of the tolerant rootstocks would be a valuable tool to decipher the basis of *R*. *necatrix* tolerance in this species.

## Methods

### Plant material and experimental design

Experiments were carried out on one-year old plants (50–60 cm height) of two avocado rootstocks, BG83 and ‘Dusa’, with contrasting disease reactions to *R*. *necatrix*. The genotype BG83 was identified as tolerant in the ongoing breeding programme at IFAPA-Málaga, while ‘Dusa’ has been previously described as susceptible to *R*. *necatrix* [5]. BG83 explants were established *in vitro* and micropropagated according to protocol [46], while plants of ‘Dusa’ were vegetatively propagated by Brokaw nursery (Spain) using a modified Frohlich method [47]. To carry out the inoculation assay with *R*. *necatrix*, plants of each rootstock (BG83 and ‘Dusa’) were grown in a greenhouse under day light illumination and semi-controlled conditions of air temperature (T) and relative humidity (RH). Photosynthetic photon flux density (PPFD), T and RH conditions inside the greenhouse were continuously registered by a quantum sensor (Apogee SQ-110, USA) and by a T/RH U23-001 HOBO Pro v2 logger (Onset Computer Corporation, USA). Maximal midday values of PPFD varied between 440 and 1012 μmol m^−2^ s^−1^, and daily T was allowed to fluctuate according to external weather conditions, but its variation range inside the greenhouse was maintained between 20±10°C by an automatic cooling system and heating when necessary. The RH values inside the greenhouse were always over 40%. *R*. *necatrix* inoculum was produced on wheat seeds according to [48]. Briefly, seeds were soaked for 12 h in 250-ml Erlenmeyer flasks filled with distilled water. The flasks, each containing 100 ml of seeds, were subsequently autoclaved after excess water had been drained off. After sterilization, four fungal disks of a 2-week-old culture of *R*. *necatrix* grown on PDA were placed aseptically in each flask and incubated at 24°C in the dark for three weeks until wheat grains were homogeneously covered by *R*. *necatrix* mycelium.

From each rootstock, nine plants were used as non-inoculated (control plants), and eighteen plants were inoculated with *R*. *necatrix*. To ensure the spread of the inoculum, it was placed in eight points scattered around the stem (~3.5 cm apart) and introduced at two depths (~5 cm and ~15 cm). Plants were inoculated with 3.75 g of colonized wheat seeds per litre of substrate and monitored during disease progression until the appearance of aerial symptoms. The experiment was laid out in a randomized block design with three biological replicates per rootstock/time-point, in which each biological replicate consisted in three plants.

### Gas exchange variables

To test the effects of *R*. *necatrix* on photosynthetic performance during the first stages of disease development, gas exchange measurements were taken periodically (~4–7 d) at midmorning (1000–1100 h) on the first two fully expanded mature leaves from the top of each plant. These leaves were labelled at the onset of the experiment for repeated measurements. An open gas exchange system Li-6400 (LICOR Inc., USA) equipped with a LED-light source (6400-02B) and with a CO_2_ mixer (6400–01) to modify the incoming air’s CO_2_ concentrations was used. The operating flow rate was 500 mL min^– 1,^ and the CO_2_ partial pressure was 400 μmol CO_2_ mol^−1^ air. All measurements were performed at 22°C with a relative humidity of 50% and at a saturating photosynthetically active photon flux density (PPFD) of 1000 μmol quanta m^−2^ s^−1^. Net CO_2_ assimilation rates (*A*_N_), stomatal conductance (*g*_s_), and transpiration rates (*E*) were estimated with established equations [49]. Intrinsic water use efficiency (*A*_N_/*g*_s_) was calculated as the ratio between *A*_N_ and *g*_s_.

The effects of *R*. *necatrix* inoculation on the gas exchange variables of the avocado rootstocks during disease progression were evaluated by one-way repeated measures analysis of variance (r-ANOVA). To analyse single sampling dates, one-way ANOVA was used. Significant differences were considered at the 5% probability level unless otherwise stated. Prior to ANOVA, normality and homogeneity assumptions were tested using the Kolmogorov-Smirnov and Cochran´s C tests, respectively. When significant differences were observed, Fisher´s least significant difference (LSD) test was used to compare the mean values. Statistical analysis was performed using STATISTICA 7.0 analytical software (Statsoft Inc., USA).

### RNA extraction

The infected roots of each ‘Dusa’ plant were harvested at eight and 22 days post-inoculation, before (‘Dusa’-b) and after (‘Dusa’-a) the observation of a decrease in net assimilation rates, although the plants appeared to be healthy. Since no significant variation in any of the gas exchange parameters was observed for the tolerant genotype, infected root samples were collected at 30 days post-inoculation eight days after the observation of a decrease in the net assimilation rate in the susceptible ‘Dusa’ genotype. RNA from ground root tissue of three independent plants was extracted using the CTAB extraction method [50], a simple and efficient method for isolating RNA from pine trees, with slight modifications. The chloroform:isoamyl alcohol step was repeated 3–5 times, depending on the stability of the interphase and colour of the sample. RNA quantity and quality were determined based on absorbance ratios at 260 nm/280 nm and 260 nm/230 nm using a NanoDrop ND-1000 (Nanodrop Technologies, Inc., Montchanin, USA) spectrophotometer. RNA integrity was confirmed by the appearance of ribosomal RNA bands and lack of degradation products after separation on a 2% agarose gel and red safe staining.

### Microarray analysis

The microarray hybridizations were carried out using a custom microarray (GEO accession GPL21856) [12]. For ‘Dusa’-b and ‘Dusa’-a plants, three biological replicate hybridizations of infected vs control samples were performed, while two biological replicate hybridizations of treated vs control samples were performed for BG83. qRT-PCR analysis was used to validate gene expression in the third BG83 biological replicate sample. Six micrograms of RNA was used for first strand cDNA synthesis. Single-stranded cDNA was synthesized according to the manufacturer´s instructions using Superscript TM III reverse transcriptase (Invitrogen) in a total volume of 30 μl. First strand synthesis was primed with random nonamer (N9, InqabaBiotec, Sunnyside, South Africa) and oligo (Dt) (dT23VN, Inqaba) primers. cDNA clean-up was carried out using an RNA clean-up kit (Qiagen RNeasy MiniElute TM) to remove hydrolysed RNA. The concentration and purity of cDNA was determined using a Nanodrop ND1000. Sample preparation and labelling for the microarray was carried out as previously described [12]. The yield and specific activity were calculated, and hybridization was performed according to the two-colour microarray-based gene expression analysis protocol (Agilent). Microarray slides were scanned using the Axon GenePix 4000B scanner (Molecular Devices, CA, USA), and Axon GenePix 6.0 software (molecular devices) was used for image assessment. GenePix Array List (GAL) files were generated by Agilent and loaded into GenePix to link information of each printed spot to analyse results. Following automated spot detection using the software, manual feature alignment was performed to validate spot finding. Flagging of features was based on saturation and signal-to-noise ratios (SNR). Features with SNR <2 in both channels were excluded from further analysis, as were features with foreground saturation >20%.

Statistical analysis of the microarray data was performed as previously described [12] using the LIMMA (linear models for microarray data) package in the R version 3.1.0 environment (R Foundation for Statistical Computing). P-values were adjusted for multiple testing using the false discovery rate (FDR) correction. A standard pair-wise Pearson correlation (r) was performed using normalized M-values to determine concordance between biological replicates. Targets were defined as differentially expressed genes (DEG) if the log2 ratio was greater or equal to 1 or smaller or equal to -1 (log_2_ ratio ≤1 and log_2_ ratio ≥ 1) and the adjusted P-value was less than or equal to 0.05 (P ≤ 0.05). The data from this study are available from the NCBI Gene Expression Omnibus under accession number GSE119140.

### Functional annotation and clustering

Functional annotation, functional enrichment and gene ontology (GO) terms describing biological processes, molecular functions and cellular components were assigned using Blast2GO software (B2G: http://www.blast2go.com). Default parameters were used with a cut-off FDR of 0.025. Venn diagrams were drawn using the Venn Diagram package [51]. Hierarchical clustering was performed using the expression values to identify genes with similar profiles across all three biological samples. Pearson correlation and linkage methods were used with the *hclust* function from the stats package [52] and the results plotted with heatmap.2 from the gplots package [53] and R color Brewer [54]. The row (contigs) dendrogram was then divided into distinct clusters, each of which contained genes with a unique expression profile by using the cutree function from the stats package [52]. Principal component analysis was performed using FactomineR [55], factoextra [56] and ade4 [57].

### Quantitative real-rime PCR

Validation of gene expression levels obtained from the microarray analysis was performed using qRT-PCR.

One microgram of total RNA was treated with DNase RNase-free (Promega, Madison, USA) following the manufacturer’s instructions. Single-stranded cDNA was synthesized using the iScript cDNA synthesis kit (BIO-RAD, California, USA) following the manufacturer´s instructions. The cDNA was analysed for genomic DNA contamination by PCR using gene-specific primers F3H-F (5´-TCTGATTTCGGAGATGACTCGC-3´) and F3H-R (5´-TGTAGACTTGGGCCACCTCTTT-3´), which flank an intron of the eflavonone 3-hydroxylase (F3H) gene. PCR amplifications were carried out as previously described by Engelbrecht and van den Berg (2013) using first-strand cDNA as the template.

The expression of fifteen avocado genes was investigated. Two endogenous control genes (actin and 18S) were used for normalization. Primer sequences for endogenous control genes and the fifteen avocado genes are presented in S3 Table. Primer pairs were chosen to generate fragments between 50 and 150 bp with melting temperatures ranging from 55–60°C and designed using Primer 3 software (http://bioinfo.ut.ee/primer3-0.4.0/).

Primer specificity was tested by first performing a conventional PCR and confirmed by the presence of a single melting curve during qRT-PCR. Serial dilutions (1:10, 1:20, 1:50, 1: 200) were made from a pool of cDNA from all treatment groups, and time-points and calibration curves were performed for each gene. For qRT-PCR, the reaction mixture consisted of cDNA first-strand template, primers (500 nmol final concentration) and SYBR Green Master Mix (SsoAdvanced Universal SYBR Green Supermix, Bio-Rad) in a total volume of 20 μl. The PCR conditions were as follows: 30 s at 95°C, followed by 40 cycles of 10 s at 95°C and 15 s at 60°C. The reactions were performed using an iQ5 real-time PCR detection system (Bio-Rad). Relative quantification of the expression levels for the target was performed using the comparative Ct method [58]. Three biological replicates of infected vs. control samples were performed for ‘Dusa’-b and ‘Dusa’-a and BG83, and all reactions were performed in triplicate. Statistical significance of the data was determined by Student´s *t*-test carried out with Sigma Stat version 4.0 software (Systat Software GmbH).

## Supporting information

S1 Table Annotation and expression values of differentially expressed genes grouped by hierarchical clustering (HCL). (XLSX)

S2 Table Top 20 overexpressed genes in ‘Dusa’-b, ‘Dusa’-a and BG83 avocado rootstocks after infection with *R*. *necatrix*. Description and features of the top 20 overexpressed genes in the ‘Dusa’-b, ‘Dusa’-a and BG83 avocado rootstocks after infection with *R*. *necatrix*.
(DOC)

S3 Table qRT-PCR primer sequences used in this study. Primer sequences used in this study.
(DOC)

S1 Fig Gene Ontology (GO) enrichment analysis of group 1 obtained in the hierarchical clustering analysis of Fig 4. Enrichment of GO terms obtained with Blast2GO using a cut-off of 0.025. (BP) biological process and (MF) molecular function.
(TIF)

S2 Fig Gene Ontology (GO) enrichment analysis of group 3 obtained in the hierarchical clustering analysis of Fig 4. Enrichment of GO terms obtained with Blast2GO using a cut-off of 0.025. (BP) biological process and (MF) molecular function.
(TIF)
