# Supplementary material for: Rosellinia necatrix infection induces differential gene expression between tolerant and susceptible avocado rootstocks
Source: PLoS One. 2019 Feb 14;14(2):e0212359. doi: 10.1371/journal.pone.0212359 (PMC6375617; doi:10.1371/journal.pone.0212359)
Supplement: S2 Table — Description and features of the top 20 overexpressed genes in the ‘Dusa’-b, ‘Dusa’-a and BG83 avocado rootstocks after infection with R. necatrix. (DOC) [file pone.0212359.s002.doc]

**S2 Table. Top 20 overexpressed genes in ‘Dusa’-b, ‘Dusa’-a and BG83 after infection with *Rosellinia necatrix*.**

| ‘Dusa’-b | | | ‘Dusa’-a | | | BG83 | | |
| --- | --- | --- | --- | --- | --- | --- | --- | --- |
| **Name** | **Description** | **Feature** | **Name** | **Description** | **Feature** | **Name** | **Description** | **Feature** |
| Pa_Contig07140 | Pathogenesis-related protein pr-4-like | Defense response | Pa_Contig04808 | Protein hothead-like | Oxidoreductasa activity | Pa_Contig06358 | ---NA--- |  |
| Pa_Contig03461 | Glucan endo- -beta-glucosidase-like | Hydrolase. Fungus cell wall degradation | Pa_Contig07184 | Protein hothead-like | Oxidoreductasa activity | Pa_Contig05213 | Glu protease inhibitor-like | Protease inhibitor |
| Pa_Contig00542 | Beta- -glucanase | Hydrolase. Fungus cell wall degradation | Pa_Contig01014 | Basic endochitinase-like partial | Fungus cell wall degradation | Pa_Contig04808 | Protein hothead-like | Oxidoreductasa activity |
| Pa_Contig07403 | Pathogenesis-related protein | Defense response | Pa_Contig00535 | Chitinase 1-like | Fungus cell wall degradation | Pa_Contig06808 | Basic 7s globulin-like (aspartic peptidase domain containing protein) | Response to salt stress |
| Pa_Contig01014 | Basic endochitinase-like partial | Fungus cell wall degradation | Pa_Contig00542 | Beta-glucanase | Fungus cell wall degradation | Pa_Contig04097 | Trypsin inhibitor | Protease inhibitor |
| Pa_Contig00535 | Chitinase 1-like | Fungus cell wall degradation | Pa_Contig00205 | Protein hothead-like | Oxidoreductasa activity | Pa_Contig06176 | Protein hothead-like | Oxidoreductasa activity |
| Pa_Contig01450 | Thaumatin-like protein | Defense response | Pa_Contig01450 | Thaumatin-like protein | Defense response | Pa_Contig02817 | Basic 7s globulin-like (aspartic peptidase domain containing protein) | Response to salt stress |
| Pa_Contig07184 | Protein hothead-like | Oxidoreductase activity | Pa_Contig03461 | Glucan endo- -beta-glucosidase-like | Hydrolase. Fungus cell wall degradation | Pa_Contig00559 | ---NA--- |  |
| Pa_Contig01608 | Cytochrome p450 89a2-like | Oxidoreductase activity | Pa_Contig06015 | Beta-D-galactosidase | Hydrolase. Fungus cell wall degradation | Pa_Contig07184 | Protein hothead-like | Oxidoreductasa activity |
| Pa_Contig01569 | Peroxidase n1-like | Oxidation/reduction process | Pa_Contig06358 | ---NA--- |  | Pa_Sin_HA66E9C01BVIYG | ---NA--- |  |
| Pa_Sin_HA66E9C01BFEF6 | Glutathione s-transferase | Detoxication and toxification mechanisms | Pa_Contig00559 | ---NA--- |  | Pa_Contig00520 | Gdsl esterase lipase at3g26430-like | Hydrolase activity, hyperosmotic salinity response. |
| Pa_Contig05982 | Pathogenesis-related protein pr-4-like | Defense response | Pa_Contig05561 | Protein hothead-like | Oxidoreductasa activity | Pa_Contig00205 | Protein hothead-like | Oxidoreductasa activity |
| Pa_Contig03623 | Cytochrome p450 93a3-like | Oxidoreductase activity | Pa_Contig00472 | Glycosyl hydrolase family protein with chitinase insertion domain | Fungus cell wall degradation | Pa_Contig01569 | Peroxidase n1-like | Oxidation/reduction process |
| Pa_Contig02847 | Cytochrome p450 89a2-like | Oxidoreductase activity | Pa_Contig05393 | Basic 7s globulin-like (aspartic peptidase domain containing protein) | Response to salt stress | Pa_Contig04419 | Basic 7s globulin-like (aspartic peptidase domain containing protein) | Response to salt stress |
| Pa_Contig01462 | Thaumatin-like protein 1 | Defense response | Pa_Contig06808 | Basic 7s globulin-like (aspartic peptidase domain containing protein) | Response to salt stress | Pa_Contig00554 | Cinnamoyl- reductase 1-like | Oxidoreductasa activity, lignin biosynthetic process. |
| Pa_Contig06176 | Protein hothead-like | Oxidoreductase activity | Pa_Contig07140 | Pathogenesis-related protein pr-4-like | Defense response | Pa_Contig02492 | Glutamate dehydrogenase 2 | Oxidation/reduction process |
| Pa_Contig06808 | Basic 7s globulin-like (aspartic peptidase domain containing protein) | Response to salt stress | Pa_Contig01063 | Pathogen-related | Defense response | Pa_Contig01652 | Cytochrome p450 71a1-like | Oxidoreductasa activity |
| Pa_Contig01093 | Chitinase 1-like | Fungus cell wall degradation | Pa_Contig02817 | Basic 7s globulin-like (aspartic peptidase domain containing protein) | Response to salt stress | Pa_Contig02540 | Tumor-related protein | Endopeptidase inhibitor |
| Pa_Contig05393 | Basic 7s globulin-like (aspartic peptidase domain containing protein) | Response to salt stress | Pa_Contig02874 | Germin-like protein subfamily 1 member 20-like | Response to salt stress, cell wall deposition | Pa_Contig02863 | Basic 7s globulin-like (aspartic peptidase domain containing protein) | Response to salt stress |
| Pa_Contig02861 | Aldo-keto reductase family 4 member c9-like | Oxidoreductase activity | Pa_Contig01462 | Thaumatin-like protein 1 | Defense response | Pa_Contig00582 | btb poz and taz domain-containing protein 1-like | Response to salt stress, response to wounding |

NA: Non Annotated
