# Supplementary material for: Rosellinia necatrix infection induces differential gene expression between tolerant and susceptible avocado rootstocks
Source: PLoS One. 2019 Feb 14;14(2):e0212359. doi: 10.1371/journal.pone.0212359 (PMC6375617; doi:10.1371/journal.pone.0212359)
Supplement: S3 Table — Primer sequences used in this study. (DOC) [file pone.0212359.s003.doc]

**S3 Table. qRT-PCR primer sequences used in this study.**

| **Contig** | **Description** | **Primer sequence** |
| --- | --- | --- |
| Actin |  | 5´-GAATCTGGACCATCTATTG-3´  5´-TACCAACCAAACCAAATC-3´ |
| 18S |  | 5´-GTCAAAGTGAAGAAATTC-3´  5´-AATCTCGTTAATCCATTC-3´ |
| Pa_Contig00456 | Methionine gamma-lyase-like | 5´-ACCCTCAGCATGCACTTCTC-3´  5´-GTTCGCCTTCTCCTCAGTCC-3´ |
| Pa_Contig02033 | Glutamine amidotransferase | 5´-CCATGGTGTTCTCCTCTGTGAA-3´  5´-CGTGCAACCGTCTGATCTCT-3´ |
| Pa_Contig03267 | Peroxidase 5-like | 5´-GGCCTCATTCGCATGCATTT-3´  5´-TTTGCCGGGGAATCCTTCTC-3´ |
| Pa_Contig02874 | Germin-like protein subfamily 1 member 20-like | 5´-TTGTCTTCCCAATTGGCCTCA-3´  5´-GGGTTCTGACTGTTGAGAGCA-3´ |
| Pa_Contig05917 | Germin-like protein | 5´-GTCAGAATGCAGGcGTTATCAC-3´  5´-TTGAAAGGCCTTGGCCAGAA-3´ |
| Pa_Contig01404 | Putative plant syntaxin | 5´-TGTGGCCCATGTTGCATACT-3´  5´-ACGATGATACCCAGAACGACG-3´ |
| Pa_Contig00951 | Kynurenine formamidase-like | 5´-CTGGGTCTCCTCAAGTGCTG-3´  5´-CCTCTCTGTAGACGTGGGAGA-3´ |
| Pa_Contig05854 | Protein hothead-like | 5´-GGTACTTGGGGTTGGTTCACT-3´  5´-TTAGGGTCGCCTGGGGATTA-3´ |
| Pa_Contig04185 | Defensin j1-2-like | 5´-GCTGCTCGTTCTTCTTCTCCT-3´  5´-ATGGAAGCCTTCAATCCTGCA-3´ |
| Pa_Contig00651 | 12-oxophytodienoate reductase 11 | 5´-GGTAGTTGAGGCCGTTGTCA-3´  5´-CTGGGTTCGAGTCTACAGCG-3´ |
| Pa_Contig00931 | Receptor protein kinase ZmPK1 | 5´-AGAGCTTGTCAAGGGAAGCC-3´  5´-GCTTCTCAGTCCCACCTCTTC-3´ |
| Pa_Contig03380 | Conserved hypothetical protein | 5´-ACCTCCATCGCTCCCTAAGT-3´  5´-ACACGGCCCAACAGAAGTAG-3´ |
| Pa_Contig05213 | Glu proteasa inhibitor | 5´-CCCAAATCGTCACGGAAGGA-3´  5´-ACAATGCCTGCAGTCTCATCA-3´ |
| Pa_Contig02540 | Tumor related protein | 5´-GCGTGTGCAGTTTGTGTAGG-3´  5´-CAAACCCAACCATCTGTTCC-3´ |
| Pa_Contig04097 | Trypsin inhibitor | 5´-TTCAATGTGCTTCTGGTGGCT-3´  5´-TTCGCCATCATCCCATCACC-3´ |

The melting temperature is 60ºC and the amplicon lengths are smaller than 150 bp.
