# Supplementary figures and images for: Rosellinia necatrix infection induces differential gene expression between tolerant and susceptible avocado rootstocks
Source: PLoS One. 2019 Feb 14;14(2):e0212359. doi: 10.1371/journal.pone.0212359 (PMC6375617; doi:10.1371/journal.pone.0212359)

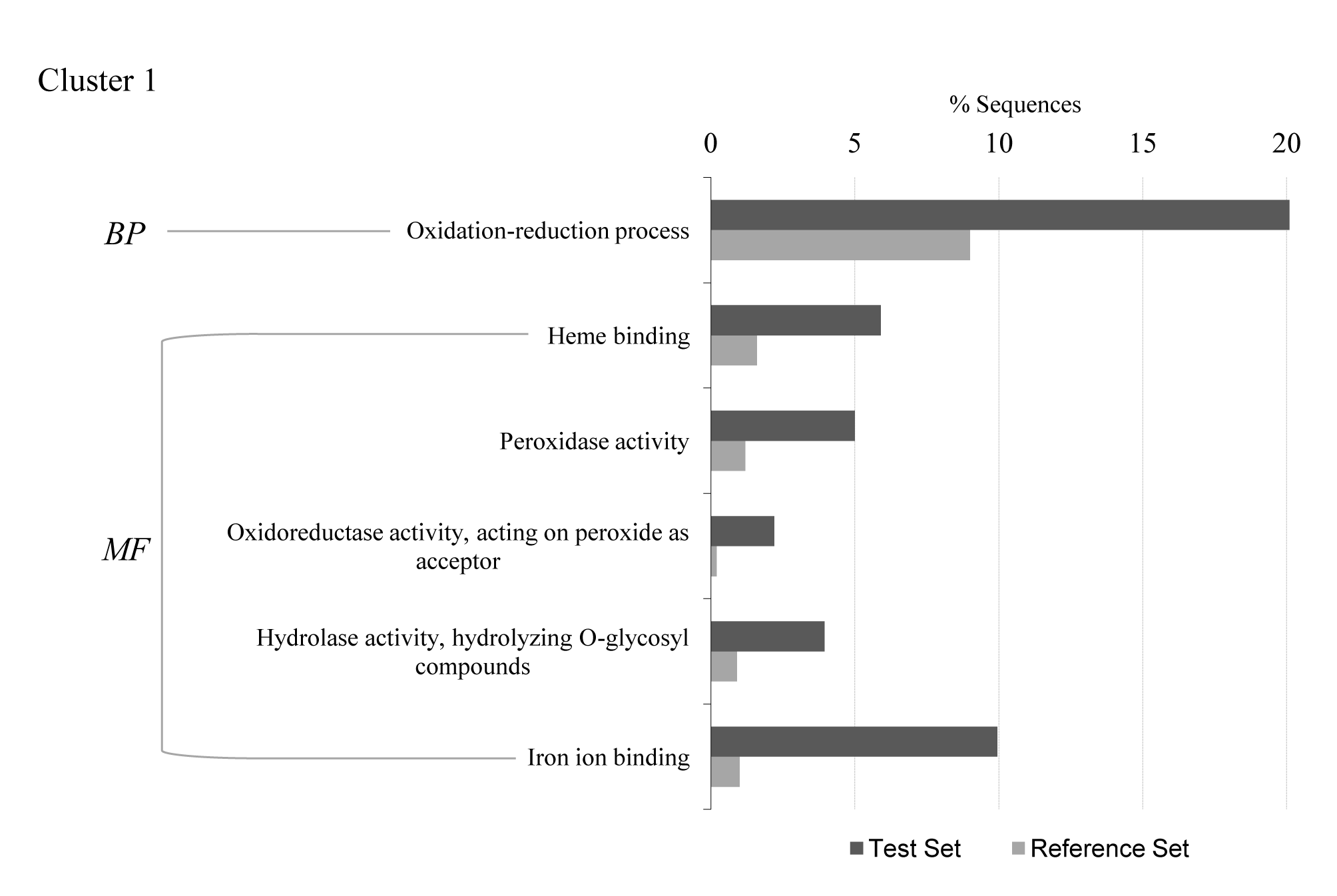

Supplement: S1 Fig — Enrichment of GO terms obtained with Blast2GO using a cut-off of 0.025. (BP) biological process and (MF) molecular function. (TIF) [file pone.0212359.s004.tif]

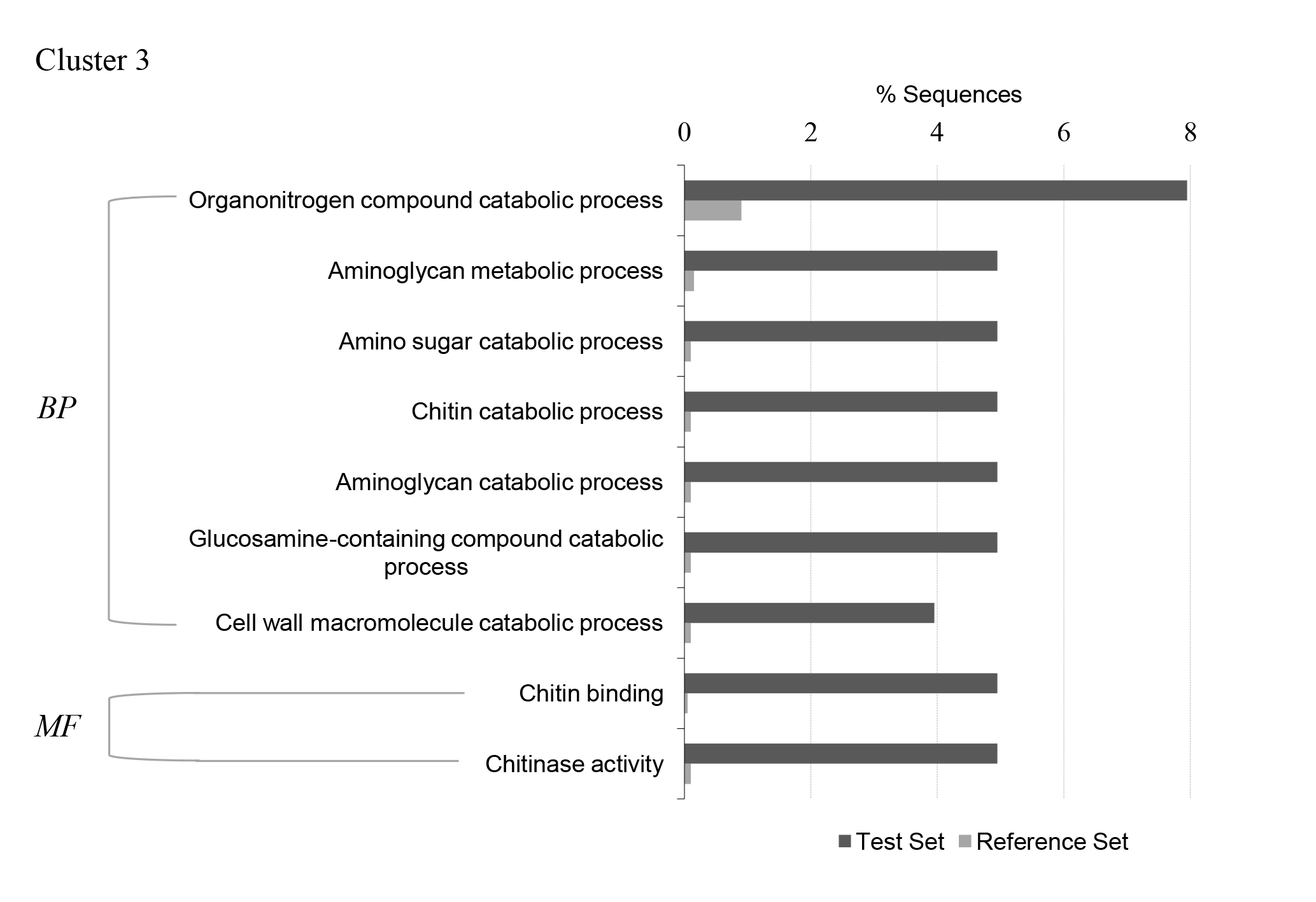

Supplement: S2 Fig — Enrichment of GO terms obtained with Blast2GO using a cut-off of 0.025. (BP) biological process and (MF) molecular function. (TIF) [file pone.0212359.s005.tif]
